# Reliability of four tests to assess body posture and the range of selected movements in individuals with spinal muscular atrophy

**DOI:** 10.1186/s12891-018-2389-8

**Published:** 2019-02-07

**Authors:** Agnieszka Stępień, Maria Jędrzejowska, Katarzyna Guzek, Witold Rekowski, Jolanta Stępowska

**Affiliations:** 1 Faculty of Rehabilitation, Józef Piłsudski University of Physical Education, Marymoncka 34, 00-968 Warsaw, Poland; 2 Mossakowski Medical Research Centre, Polish Academy of Sciences, Neuromuscular Unit, Pawińskiego 5, 02-106 Warsaw, Poland

**Keywords:** Spinal muscular atrophy, Reliability, Scoliosis, Chest deformity, Pelvic obliquity, Cervical rotation, Hip mobility

## Abstract

**Background:**

The majority of individuals with spinal muscular atrophy (SMA) experience progressive skeletal deformities which may affect the quality of life and mobility. To date, no studies have evaluated the reliability of tests assessing body posture and joint mobility in SMA patients. The purpose of this study was to assess the reliability of Cervical Rotation test (CR), Supine Angle of Trunk Rotation test (SATR), Hip Extension test (HE) and Pelvic Obliquity test (PO) developed to evaluate the musculoskeletal system in SMA individuals.

**Methods:**

Thirty individuals (12 girls and 18 boys) aged 4–15 with SMA type II (*n* = 24) and III (*n* = 6) confirmed by genetic examinations were qualified for the study. The participants were examined twice by three physiotherapists on the same day. The examination included four tests, i.e. CR, SATR, HE and PO tests aimed at assessing ranges of rotation in the cervical spine, chest deformities, ranges of hip extension and pelvis position while sitting. Statistical calculations were made with the use of statistical software IBM SPSS Statistics version 20. Reliability was assessed using the Intraclass Correlation Coefficient (ICC).

**Results:**

Intraobserver reliability was excellent for CR (ICC range 0.839–0.911), SATR (ICC range 0.918–0.939 - the upper part of the sternum; ICC range 0.951–0.975 - the lower part of the sternum), HE (ICC range 0.988–0,991) and PO (ICC range 0.896–0.935) tests.

The interobserver ICC reached the excellent values in CR (ICC range 0.912–0.920), SATR (ICC = 0.888 - the upper part of the sternum, ICC = 0.951 - the lower part of the sternum), HE (ICC range 0.922–0.923) and PO (ICC = 0.928) tests.

**Conclusions:**

CR, SATR, HE and PO tests are reliable and may be used for examining individuals with SMA. The application of these tests provides a possibility to detect early changes in the musculoskeletal system in children and adolescents and to assess the effectiveness of the implemented pharmacotherapy and rehabilitation.

## Background

Spinal Muscular Atrophy (SMA) is a rare genetic disease related to the mutations of SMN1 gene which manifests itself with a progressive impairment and atrophy of muscles resulting from the loss of motor cells of the spinal cord [1, 2]. The majority of patients experience limitations in the activities of daily living, progressive skeletal deformities, range of motion limitations and deterioration in breathing parameters [3–12].

Proper functional assessment of patients and early implementation of treatment may lengthen life and improve the quality of life [7, 8, 13]. Various functional scales [14–20] and the Six-Minute Walk Test [21] are used to assess motor abilities of individuals with SMA. Moreover, ranges of motion in limbs and muscle strength are measured, while breathing capability test and X-Ray examination serve as a valuable completion of the evaluation [4, 6, 7, 22, 23].

No studies have evaluated the reliability of tests assessing body posture and joint mobility in SMA patients, despite the fact they experience numerous skeletal deformities. One of the symptoms typical of SMA patients is scoliosis which is accompanied by improper head position, chest deformity and oblique position of pelvis [3, 8, 9, 12]. Difficulties in treating scoliosis are also caused by hip joint dislocations [12, 24] and increasing contractures [7, 10, 23].

Although in the literature we can find numerous studies regarding the diagnostics and treatment of idiopathic scoliosis, there are few references to the assessment and treatment of patients with neuromuscular scoliosis. A radiological examination is recognized as the objective tool for assessing the spine, pelvis and hip joints in patients with neuromuscular scoliosis. Monitoring is recommended at least once a year, mainly in individuals with scoliosis and those who have lost the ability of ambulation [13, 25].

The method to detect early symptoms of spinal, chest, pelvic and hip disorders without an X-ray image has not been described. Measurement of the angle of trunk rotation recommended for patients with idiopathic scoliosis and applied in screening studies [26, 27] cannot be used in the majority of SMA patients due to difficulties in maintaining a sitting and standing position or bending forward. The lack of proper tests assessing postural parameters in everyday clinical practice limits also a possibility of monitoring changes in the musculoskeletal system in SMA patients with scoliosis and makes it difficult to plan proper treatment and document the effectiveness of the applied procedures.

Therefore, we decided to put forward tests used in our daily practice to examine the cervical spine, chest, pelvis and hip joints. We observe that limitation of cervical rotation, chest deformity, pelvic obliquity and uneven ranges of motion in the hip joints are often related to scoliosis in SMA patients. These impairments may affect the basic vital functions such as breathing and swallowing, and maintaining the body against gravity. The proposed tests don’t give information about all progressive deformations in the skeletal system. None of the tests is intended for direct assessment of scoliosis and hip dislocation.

The aim of this study was to determine the intra- and interrater reliability of four measurements: Cervical Rotation test (CR), Supine Angle of Trunk Rotation test (SATR), Hip Extension test (HE) and Pelvic Obliquity test (PO).

Some of these tests have been used in the past by other researchers, but their reliability has not been studied in SMA patients.

## Methods

### Participants

Patients with SMA type II and type III confirmed genetically, aged 4–15, were qualified for the study. Individuals after spine or hip surgery, patients who could not lie flat on their backs or sit without assistance were not qualified. This study recruited the total of 30 SMA patients participating in the conference and workshops organised by the SMA Foundation for patients and their families. Parents of all participants signed the consent form to participate. This study was approved by the Senate Ethics Committee of the University of Physical Education in Warsaw (SKE 01–03/2016).

### Study protocol

The research was conducted by three physiotherapists with several years of experience in working with SMA individuals. Each of them carried out the examination independently according to the established protocol. Prior to the study, the physiotherapists had undergone training regarding the performance of CR, SATR, HE and PO tests included in the protocol.

Physiotherapists performed all the tests in each of the study participants in separate locations in two separate rounds with at least 30-min intervals. After finishing the first round, the measurements were repeated in the second round. In total, each physiotherapist performed two measurements in each of the tests in each participant. During the intervals the participants rested. Each examiner was blinded to the previous measurements and the measurements of other two observers. The results were recorded in separate forms.

The SATR and PO tests were conducted with the use of a scoliometer (Orthopedic Systems Inc. OSI 1995) while the measurements in CR and HE tests were performed with the use of Rippstein’s plurimeter (Rippstein, Switzerland). Values in degrees were obtained. Previous studies have confirmed the reliability of measurements of trunk position with the use of a scoliometer [28–30] and the measurements of ranges of motion with the use of a plurimeter [31–33]. The performance of CR, SATR, HE and PO tests is presented below.

#### Cervical rotation test (CR)

**Fig. 1:**
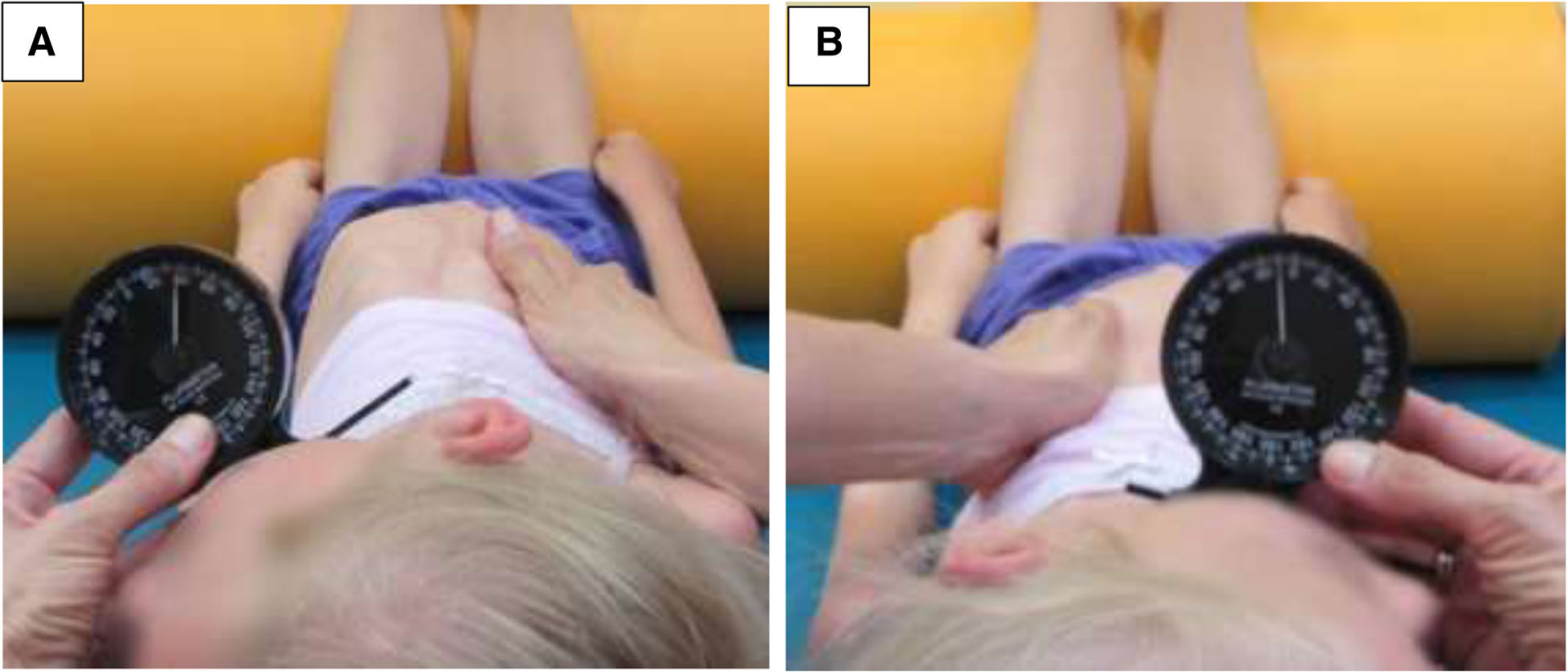
Cervical Rotation test (CR): **a** rotation to the left, **b** rotation to the right

The range of passive rotation of the cervical spine was assessed in a supine position with upper limbs parallel to the sides of the body. Patients with contractures in elbows had their forearms supported. Lower limbs were slightly bent and a roll was placed under knees. The physiotherapist helped a patient to perform head rotation (without lateral flexion) at the same time stabilising ribs in the sternal area on the side opposite to the direction of rotation. The Rippstein’s plurimeter was reset to zero in relation to the surface. The arm of the plurimeter was placed on the corpus mandibulae (Fig. 1a, b). The degree values of inclination above the level were marked with “-” (limitation of rotation), while those below the level were marked with “+”.

#### Supine angle of trunk rotation test (SATR)

**Fig. 2:**
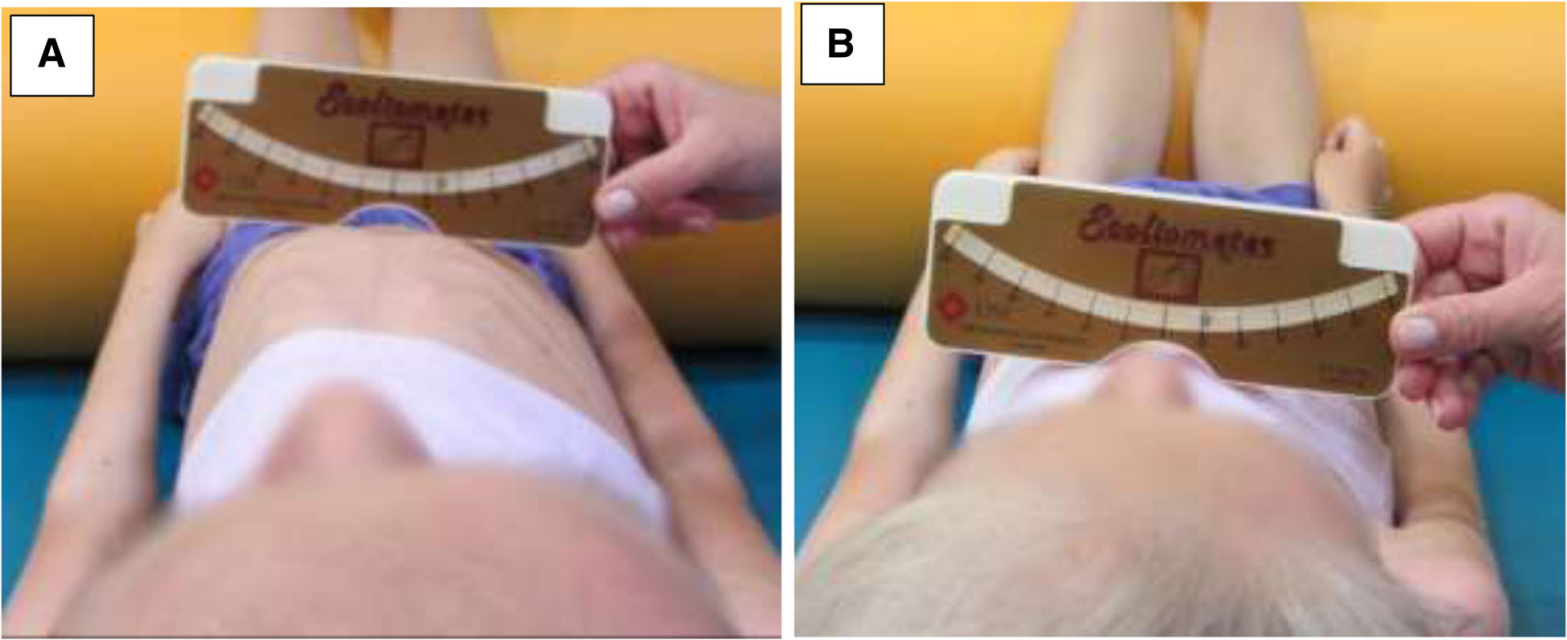
Supine Angle of Trunk Rotation test (SATR): **a** an upper part of the sternum (SATRU), **b** a lower part of the sternum (SATRL)

The measurement of the angle of trunk rotation was performed in a supine position. Head was kept symmetrically. The scoliometer was placed on the sternum and ribs perpendicularly to the longitudinal body axis in two places, i.e. at the 2nd-3rd rib below the sternal angle (SATR upper – SATRU) (Fig. 2a) and in the lower part of the sternum, where corpus sterni connects with processus xiphoideus (SATR lower – SATRL) (Fig. 2b).

#### The hip extension test (HE)

**Fig. 3:**
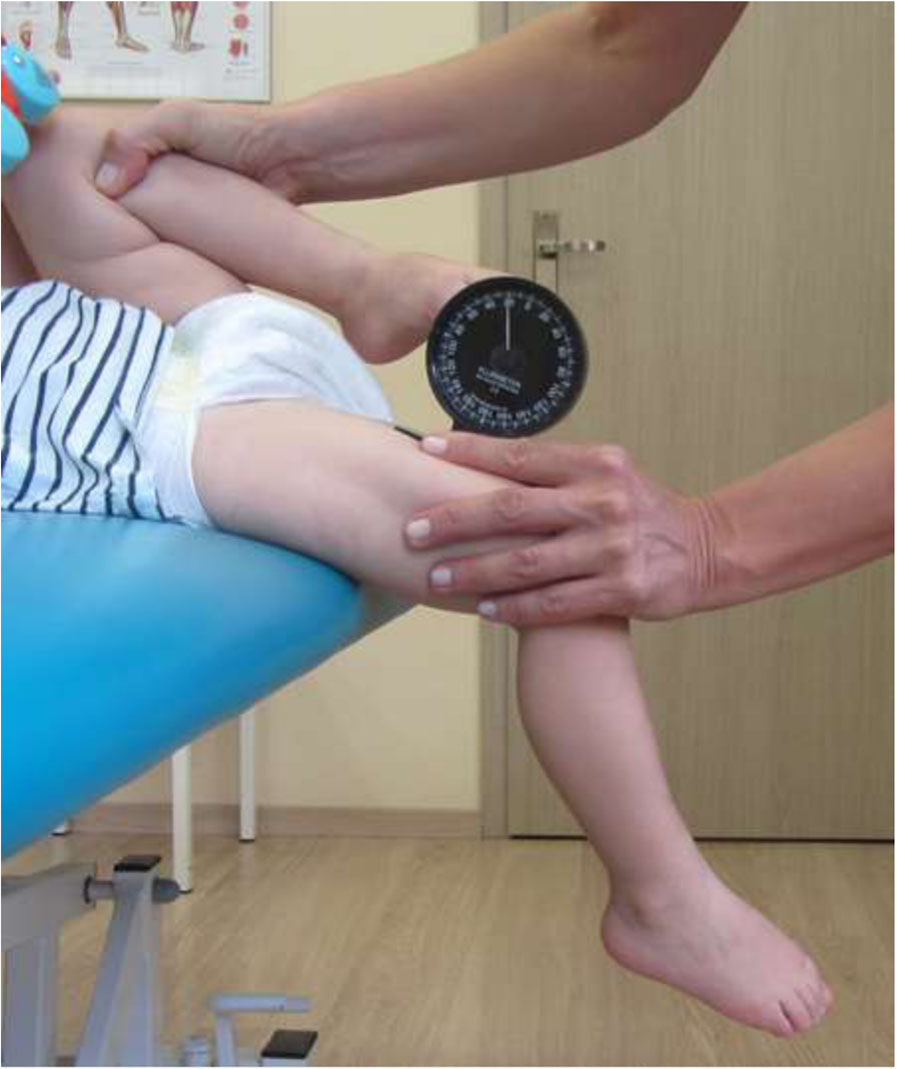
Hip Extension test (HE) – the range of hip extension in the right hip

The passive range of hip extension was measured in a standard supine position [34]. First, the lower limbs were flexed towards the chest until the sacral bone moved. Then, one limb was stabilised, while the other one was lowered below the edge of the table. The Rippstein plurimeter in a starting position parallel to the floor was placed on the thigh above the base of patella (Fig. 3). The values of extension below the level were marked with “+”, while the values above zero (limitation of extension) were marked with “-”.

#### Pelvic obliquity test (PO)

**Fig. 4:**
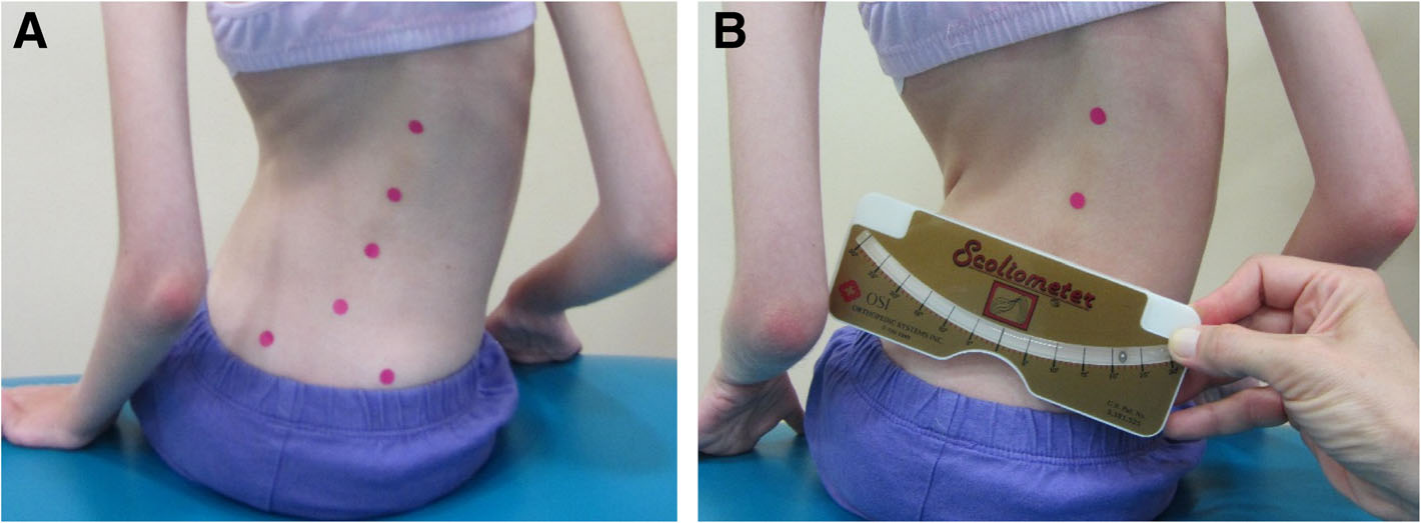
Pelvic Obliquity test (PO): **a** Points marked on the posterior superior iliac spines, **b** The measurement of the oblique pelvis position

PO test was performed in a sitting position with supported lower limbs. The physiotherapists marked points on the posterior superior iliac spines (Fig. 4a) and then placed a lower edge of the scoliometer on the points (Fig. 4b).

### Statistical analyses

Statistical calculations were made with the use of statistical software IBM SPSS Statistics version 20. The means, standard deviations, interclass correlation coefficient (ICC) [35] and 95% confidence intervals were calculated. When assessing reliability, the following criteria were taken into account: the coefficient value lower than 0.40 – poor reliability, between 0.40 and 0.59 – fair reliability, between 0.60 and 0.74 – good reliability, between 0.75 and 1.00 – excellent reliability [36]. The level of significance was set at 0.05.

## Results

The study group of 30 participants included 24 individuals with SMA type II and 6 with SMA type III aged 4–15 (mean age 7.9 ± 3.1, body mass 25.0 ± 9.8, body height 125.2 ± 16.3). There were 12 girls and 18 boys. At the time of the examination, 3 of the 6 patients with type III SMA were able to walk. In 14 individuals, scoliosis had been diagnosed. In one case, the results of PO test were not included in the statistical analysis due to imbalance in the sitting position during measurements.

Intraobserver reliability was excellent in all the tests. High values of the ICC, ranging between 0.839 and 0.991, were obtained. The lowest value of the ICC was noted in the CR test, while the highest value was noted in the HE test (Table 1).

**Table 1:** Intraobserver reliability for CR, SATR, HE, PO tests - intraclass correlation coefficient (ICC) and 95% confidence intervals

Test	Observer	ICC for one observer	95% confidence interval
CRleft	1	0.901	0.802–0.951
	2	0.891	0.784–0.947
	3	0.839	0.689–0.920
CRright	1	0.908	0.817–0.955
	2	0.904	0.809–0.953
	3	0.911	0.822–0.957
SATRU	1	0.920	0.839–0.961
	2	0.918	0.834–0.960
	3	0.939	0.877–0.971
SATRL	1	0.975	0.949–0.988
	2	0.951	0.900–0.977
	3	0.951	0.900–0.976
HEleft	1	0.990	0.979–0.995
	2	0.988	0.974–0.994
	3	0.991	0.980–0.998
HEright	1	0.988	0.975–0.994
	2	0.989	0.978–0.995
	3	0.991	0.980–0.955
PO	1	0.929	0.855–0.966
	2	0.896	0.791–0.950
	3	0.935	0.867–0.969

*ICC* intraclass correlation coefficient, *CRleft/right* cervical rotation to left/right, *SATRU* anterior angle of trunk rotation upper part, *SATRL* anterior angle of trunk rotation lower part, *PO* pelvic oblique, *HEleft/right* hip extension on the left/right

Interobserver reliability was also excellent, which was indicated by the value of ICC ranging between 0.888 and 0.993 in CR, SATR,HE and PO tests. The lowest value of the ICC was noted in SATRU test, while the highest value was observed in HE test (Table 2).

**Table 2:** Interobserver reliability for CR, SATR, PO, HE tests - intraclass correlation coefficient (ICC) and 95% confidence intervals

Test	ICC for three observers	95% confidence interval
CRleft	0.912	0.846–0.954
CRright	0.920	0.807–0.941
SATRU	0.888	0.738–0.917
SATRL	0.951	0.912–0.974
HEleft	0.993	0.988–0.997
HEright	0.992	0.986–0.996
PO	0.928	0.873–0.963

*ICC* intraclass correlation coefficient, *CRleft/right* cervical rotation to left/right, *SATRU* anterior angle of trunk rotation upper part, *SATRL* anterior angle of trunk rotation lower part, *PO* pelvic oblique, *HEleft/right* hip extension on the left/right

Mean values obtained in CR, SATR, HE and PO tests are included in Table 3.

**Table 3:** Mean values obtained in CR, SATR, HE and PO tests

Test	Mean values /one observer (^o^) ± SD	Mean values / three observers (^o^) ± SD
CRleft	1. 22.7 ± 6.0	
	2. 22.2 ± 6.5	22.2 ± 6.0
	3. 21.7 ± 5.6	
CRright	1. 21.9 ± 6.1	
	2. 21.4 ± 5.9	21.5 ± 6.0
	3. 21.3 ± 6.0	
SATRU	1. 2.8 ± 2.0	
	2. 2.4 ± 1.8	2.5 ± 2.0
	3. 2.4 ± 2.1	
SATRL	1. 3.1 ± 3.6	
	2. 3.0 ± 3.1	3.0 ± 3.2
	3. 2.8 ± 3.0	
HEleft	1. -15.0 ± 21.6	
	2. -14.6 ± 21.1	−14.9 ± 21.2
	3. -15.1 ± 20.8	
HEright	1. -13.3 ± 21.3	
	2. -14.0 ± 21.3	−13.6 ± 21.4
	3. -13.4 ± 21.6	
PO	1. 5.0 ± 3.5	
	2. 4.5 ± 3.0	4.7 ± 3.2
	3. 4.6 ± 3.0	

*CRleft/right* cervical rotation to left/right, *SATRU* anterior angle of trunk rotation upper part, *SATRL* anterior angle of trunk rotation lower part, *PO* pelvic oblique, *HEleft/right* hip extension on the left/right, *SD* standard deviation, (^o^) degrees

## Discussion

Early detection of disorders in the musculoskeletal system makes it possible to implement appropriate prevention or treatment adapted to individual needs. It is particularly significant in children and youth who are in their development period. Individuals with SMA experience numerous disorders in the musculoskeletal system. A radiological examination is the basic method of assessment; however, there is a lack of other reliable tests to assess these disorders. The purpose of the present study was to assess the intraobserver and interobserver reliability of four tests which can be used to evaluate body posture and selected ranges of motion in SMA individuals.

The obtained results indicate that CR, SATR, HE and PO tests may be applied in the functional diagnostics of children and adolescents with SMA. It is difficult to refer the findings to other results as, to date, no studies have evaluated the reliability of similar tests in SMA patients. In the past, researchers revealed high reliability of a few functional scales and tests [14, 17, 18, 20, 21]. The reliability of the muscle strength test with the use of hand dynamometer was also assessed [22].

One of important aims of rehabilitation for individuals with SMA which are included in current recommendations is to prevent limitations in ranges of motion [13]. Two of the tests suggested in our study, i.e. CR and HE tests, are used to measure ranges of rotation in the cervical segment of the spine as well as hip extension.

Ranges of rotation in the cervical spine in SMA individuals have not been assessed by other researchers. The CR test provides a possibility to measure rotational mobility and detect compensation of scoliosis in the cervical spine. Asymmetric head position usually resulting from spinal deformities and improper pelvis position [7, 8] may lead to movement limitations in the cervical spine. Improper head position and limitation of rotational movements of the cervical spine may be one of the reasons for swallowing difficulties described in SMA patients [37].

Regular measurements of the range of hip extension are particularly significant for patients who undergo supported standing. Ranges of motion in hip joints in SMA subjects have been studied extensively. The measurements were most often performed with the use of goniometer [7, 10, 23]. Some researchers have indicated the necessity to assess mobility in limb joints and to implement stretching exercises in SMA patients due to frequent mobility limitations [8, 10, 13, 23].

Nobody has assessed the reliability of measurements of hip extension in SMA individuals. Van Dillen et al. [38] evaluated intra-rater reliability of the measurements of hip extension in a supine position with a knee bent at 80^o^ with the use of goniometer in the group of 35 healthy individuals and obtained the value of ICC between 0.70 and 0.89. Pandya et al. [39] assessed reliability of the measurements of hip extension in the group of 105 boys with Duchenne muscular dystrophy. The intra-rater reliability in this group was excellent and inter-rater reliability was good. In HE test, carried out in the same position but with the use of plurimeter, high level of both intraobserver and interobserver reliability were revealed.

A proper chest shape is one of the factors determining breathing. Deformities of the chest and improper pelvis position associated with scoliosis in SMA patients have been described in the literature. However, radiological assessment was the only diagnostic test applied in research [3, 8–10]. It is worth noting that an X-ray recommended in the case of clear symptoms of scoliosis is rarely recommended for early diagnosis of pelvic asymmetry or the first symptoms of chest deformity. The SATR and PO tests presented in this research provide such an opportunity.

The SATR test was proposed to assess deformities of the anterior part of the chest. An improper chest position is most commonly related to scoliosis, ribs deformity or an improper breathing mechanism. The SATR test makes it possible to assess chest deformity in individuals at various stages of functioning, which is particularly significant due to breathing disorders and frequent respiratory tract infections in SMA patients [4–7, 11]. The biggest angle values in the test were noted in our study in patients with scoliosis. Therefore, the SATR test may be considered as one of the intermediary tests assessing spinal deformity.

Pelvic obliquity constitutes a serious problem in orthopaedic treatment of neuromuscular scoliosis [3, 7, 8, 12]. The PO test makes it possible to detect slight symptoms of an improper pelvis position and to implement early prevention procedures.

The tests presented in this paper provide an additional opportunity to perform an initial examination of a patient and to diagnose early disorders of the musculoskeletal system. They also make it possible to assess the effectiveness of prophylactic and treatment procedures. It may be expected that new ways pharmacological treatment will change a natural course of the disease. Therefore, it seems even more significant to implement new unified methods of functional assessment in clinical practice as they objectify treatment results and allow us to detect complications at an early stage. We believe that tests presented in our manuscript, useful in everyday practice, will be a clinical supplement to the radiological diagnostics.

The most significant limitation of this study was a short interval between the first and the second series of measurements. Carrying out the tests in a longer period was not possible for organizational reasons.

## Conclusions

The CR, SATR, HE and PO tests are reliable and may be used for examining SMA patients. The tests provide an additional method in early diagnostic of some important aspects of the musculoskeletal system in order to plan physiotherapeutic procedures and to assess treatment effectiveness. It is significant to refer the values of these tests performed in the group of SMA patients to the values obtained from healthy individuals.

## Abbreviations

CR: Cervical Rotation test
HE: Hip Extension test
ICC: Interclass correlation coefficient
PO: Pelvic Obliquity test
SATR: Supine Angle of Trunk Rotation test
SATRL: Supine Angle of Trunk Rotation Lower test
SATRU: Supine Angle of Trunk Rotation Upper test
SMA: Spinal muscular atrophy
